# The Effects of Glucagon‐Like Peptide‐1 Receptor Agonists and Sodium‐Glucose Co‐Transporter‐2 Inhibitors on Lean Body Mass in Humans: A Systematic Review and Meta‐Analysis of Randomised Controlled Trials

**DOI:** 10.1002/dmrr.70194

**Published:** 2026-06-19

**Authors:** Rishi Jobanputra, Jack A. Sargeant, Tatiana Plekhanova, Emily James, Abdullah Almaqhawi, David R. Webb, Melanie J. Davies, Thomas Yates

**Affiliations:** ^1^ Diabetes Research Centre University of Leicester Leicester UK; ^2^ National Institute for Health Research (NIHR) Leicester Biomedical Research Centre Leicester UK; ^3^ Department of Family and Community Medicine, College of Medicine King Faisal University Al Ahsa Saudi Arabia

**Keywords:** body composition, glucagon‐like peptide‐1 receptor agonists, lean body mass, sodium‐glucose co‐transporter‐2 inhibitors, weight loss

## Abstract

**Aims:**

The use of pharmacotherapies with weight loss properties for the management of obesity and chronic disease are now routinely prescribed. We investigated the evidence from randomised‐controlled trials for the effects on lean body mass of glucagon‐like peptide‐1 receptor agonists (GLP‐1RA) and sodium‐glucose co‐transporter‐2 inhibitors (SGLT2i) in humans.

**Materials and Methods:**

PubMed, MEDLINE, the Cochrane Library and CINAHL were searched from inception to October 2022. The primary outcome was change in body composition focused on measures of lean body mass (LBM).

**Results:**

Thirty‐six studies in population groups with obesity (*n* = 8), type 2 diabetes mellitus (*n* = 20), type 1 diabetes mellitus (*n* = 5) and polycystic ovary syndrome (*n* = 3) receiving a GLP‐1RA or SGLT2i therapy versus placebo or active comparator satisfied our inclusion criteria, including 21 for GLP‐1RA and 15 for SGLT2i. Meta‐analysis showed an overall loss of LBM in the direction of the intervention groups prescribed GLP‐1RA (MD: −1.51 kg [95% CI: −2.00 to −1.01]) and SGLT2i (MD: −1.04 kg [95% CI: −1.45 to −0.64]) with sub‐group analysis showing largely consistent results when stratified by type of body composition outcome, type of body composition measurement technique and disease status. Results were not modified by sex. Further meta‐analysis found that lean mass accounted for 28% (95% CI: 22%–34%) of overall weight loss induced by GLP‐1RA and SGLT2i.

**Conclusions:**

GLP‐1RA and SGLT2i are associated with a loss of LBM that appears congruent with overall weight loss. Monitoring of body composition and provision of combined therapy to preserve lean mass during weight loss should be considered.

## 1 Introduction

Type 2 diabetes mellitus (T2DM) and obesity are major global health challenges [1]. Glucagon‐like peptide‐1 receptor agonists (GLP‐1RAs) and sodium‐glucose co‐transporter‐2 inhibitors (SGLT2is) combine glucose lowering with weight lowering effects and are recommended in recent guidelines for the management of type 2 diabetes mellitus alongside lifestyle interventions [2]. In addition, they are also increasingly recommended for use in wider populations, with GLP‐1RAs developed and licenced specifically for weight management, while SGLT2is are recommended for heart failure and chronic kidney disease (CKD) [3].

GLP‐1 is a gut hormone that slows gastric emptying and gut motility in response to nutrient load, thus leading to increased satiety and reductions in body weight [4, 5]. Liraglutide was the first GLP‐1RA to be approved for use by the U.S. Food and Drug Administration (FDA) and European Medicines Agency (EMA) specifically for the management of obesity. A study investigating the effect of liraglutide 3.0 mg once‐daily on body weight in a population living with obesity, showed that 63.2% of individuals assigned to the liraglutide arm lost at least 5% of their body weight, with 33.1% losing more than 10% at 56 weeks [6]. More recent evidence with once‐weekly GLP‐1RA semaglutide at 2.4 mg in adults with overweight or obesity, highlighted greater reductions in body weight, with 69.1% of participants achieving ≥ 10% weight loss and 50.5% of individuals achieving ≥ 15% weight loss at 68 weeks [7]. Additionally, the latest therapies combining GLP‐1RAs with other incretin hormones provide further advances in effectiveness, as a recent trial showed that 57% of those with obesity prescribed 15 mg tirzepatide were found to achieve 20% weight loss by week 72 [8].

SGLT2is act by inhibiting sodium glucose transporter 2 channels in the kidney tubules, thus increasing glycosuria, leading to a caloric loss [9]. It is known that inhibition of SGLT2 can lead to an excretion of around 60–100 g of glucose per day and studies have shown mean weight reductions in the region of 1.5–2 kg [10, 11]. A recent meta‐analysis showed that SGLT2is elicited significant reductions in overall body weight of −1.42 kg [12].

Whilst weight loss has many established benefits in the prevention of chronic metabolic disease, the potential impact on body composition and overall physical health status needs considering. When weight loss occurs through diet induced energy restriction, at least 25% of overall body weight loss consists of lean body mass (LBM) [13]. LBM is primarily composed of skeletal muscle aiding in movement, while acting as metabolically active tissue supporting glucose regulation and metabolic homeostasis [14]. The loss of LBM may therefore act to partially attenuate some of the wider whole body clinical benefits of weight loss and conversely, LBM preservation with weight loss is an important consideration for optimising health benefits while improving functional reserve [15]. Furthermore, individuals with T2DM have up to a five times increased risk of frailty [16]. Losses in LBM as a result of weight loss in individuals already presenting with or at high risk of frailty can lead to a reduction in functional capacity, while increasing the risks of falls and subsequent hospitalisation [17].

A previous narrative review suggested that LBM loss associated with weight loss induced by GLP‐1RAs and SGLT2is was similar to that observed with diet induced weight loss. Results showed that between 20% and 50% of overall weight loss was through loss of fat‐free mass (FFM) [18], however quantitative synthesis of the collective evidence is lacking. Establishing the overall pattern of LBM loss with GLP‐1RAs and SGLT2is will aid in identifying a potential target for future combined interventions in clinical care, focused specifically on LBM preservation. The aim of this systematic review and meta‐analysis is to quantify the effects of GLP‐1RAs and SGLT2is on LBM as stand‐alone therapies.

## 2 Materials and Methods

This systematic review and meta‐analysis was conducted in accordance with the Preferred Reporting Items for Systematic Reviews and Meta‐Analyses (PRISMA) statement [19]. The protocol is registered in the International Prospective Register of Systematic Reviews (PROSPERO; registration ID: CRD42022350621).

### 2.1 Study Eligibility and Outcomes of Interest

The study inclusion criteria consisted of randomised‐controlled trials, which investigated the effect of a GLP‐1RA or SGLT2i for the primary or secondary outcome of body composition in adults (men and women) aged ≥ 18 years. Pharmacotherapies of interest were GLP‐1RAs, SGLT2is and dual gastric inhibitory polypeptide (GIP)/GLP‐1RAs as follows: liraglutide, semaglutide, exenatide, dulaglutide, efpeglenatide, langlenatide, albiglutide, canagliflozin, dapagliflozin, empagliflozin, ipragliflozin, luseogliflozin, tofogliflozin, ertugliflozin, sotagliflozin and tirzepatide. The comparator arm consisted of placebo or T2DM medications including metformin, glimepiride, insulin, glibenclamide, tenegliptin and pioglitazone. The selection criteria for trials were the treatment intervention group had to have consisted of GLP‐1RA, SGLT2i or dual GIP/GLP‐1RA therapies and the control group had to consist of other glucose‐lowering pharmacotherapies, lifestyle intervention or placebo and measures of body composition had to include either fat‐free mass (FFM; defined as all non‐fat components of the body), lean body mass (LBM; defined as FFM minus total bone mass) or skeletal muscle mass (SMM; defined as FFM minus total bone mass, connective tissue, skin and organs). To aid readability, LBM will be used to refer to all three definitions unless specified.

The outcome of interest was body composition measured by dual‐energy x‐ray absorptiometry (DEXA), bio‐electrical impedance analysis (BIA), magnetic resonance imaging (MRI), air‐displacement plethysmography (BodPod), (ADP), hydrostatic (underwater) weighing and skinfold tests. Non‐randomised studies, observational cohort studies, studies published in a language other than English, non‐peer reviewed publications, abstracts, conference posters, government reports and unpublished studies were excluded.

### 2.2 Search Strategy

PubMed, MEDLINE, The Cochrane Library and CINAHL databases were used to search for literature published from inception to October 2022. Key subject terminologies and phrases used in the search were as follows: ‘body weight’, ‘body composition’, ‘lean muscle mass’, ‘fat free mass’, ‘GLP‐1RAs’, ‘SGLT2i’, ‘dual GIP/GLP‐1RA’. The full MEDLINE search strategy is presented in the Supporting Information S1: Methods S1. Grey literature was searched using Google Scholar.

### 2.3 Study Selection

Following the searches, all search results were exported to a reference management software [20]. Two reviewers (R.J. and A.A.) independently screened for potentially relevant articles based on the titles and abstracts. Full texts were screened by the same reviewers and any conflicts during the study selection process were resolved by consensus with a third reviewer (E.J.).

### 2.4 Data Extraction and Quality Assessment

Data extraction was conducted from eligible articles independently by two reviewers (R.J. and E.J.). Study data were extracted using a pre‐formed data extraction spreadsheet. Variables of interest included first author, publication year, sample sizes, pharmacotherapy class and doses, control group agents and participant demographics. Data extracted for the outcomes of interest consisted of body composition parameters: total body weight, total body fat percentage, LBM, FM and SMM. Outcome data were extracted as mean change and standard deviation for both the intervention arm and control/placebo. Outcome data presented in varying formats (e.g., where within group change was presented as standard error or 95% confidence interval), were calculated or estimated to standard deviation by adopting established equations from the Cochrane handbook [21]. For multi‐arm trials included in the meta‐analysis, data for the treatment arm at the maximum dose were extracted.

Quality assessment was conducted independently by two authors (R.J. and E.J.), by implementing the Risk of Bias 2 (RoB 2) assessment tool (2019 version) which was used for randomised parallel group trials and the Cochrane Risk of bias 2 (RoB 2) was used for randomised cross‐over studies [22]. The Quality assessment was conducted based on five domains: randomisation process, deviations from the intended interventions, missing outcome data, measurement of the outcome and selection of the reported result. Any discrepancies between reviewers were resolved in consensus.

### 2.5 Statistical Analysis

All the studies included in this systematic review and meta‐analysis were recorded in tables. A random effects meta‐analysis was conducted for the primary outcome, accounting for intra and inter‐study variability. Both the standardised mean difference (SMD) and weighted mean difference (WMD) along with the 95% confidence intervals (CI) were generated for the outcome of interest. Data for LBM was prioritised for inclusion as the most commonly reported outcome measure of interest, however, where LBM was not reported, FFM data were analysed and where FFM was not reported, SMM data were analysed. Results were reported separately by therapy class (GLP‐1RA and SGLT2i). To highlight sources of heterogeneity, sub‐group analysis was performed based on methods of defining body composition (LBM, FFM or SMM), methods of measuring body composition (DEXA, BIA, MRI, ADP), disease status based on primary inclusion criteria (categorised as general obesity, T2DM, T1DM or PCOS) and comparison type (placebo‐controlled or active comparator). In addition, we undertook a further meta‐analysis in the intervention group only, analysing the proportion of weight loss attributable to LBM (or available measure)—derived by dividing the change in lean mass by the change in overall weight. For descriptive purposes, this was converted to a percentage by multiplying by 100. The Higgins *I* ^2^ statistical test was used to interpret heterogeneity [23]. Studies with *I* ^2^ statistic of 25%–50% were deemed as having low heterogeneity, 50%–75% were characterised as a moderate heterogeneity and an *I* ^2^ statistic greater than 75% were deemed as a high heterogeneity. Publication bias was assessed using funnel plots. A *p* value of < 0.05 (5%) was used to denote statistical significance. Meta‐analysis was conducted using Review Manager (RevMan) version 5.4.1 and STATA/BE 18.0 for windows.

## 3 Results

### 3.1 Literature Search and Study Characteristics

**FIGURE 1:**
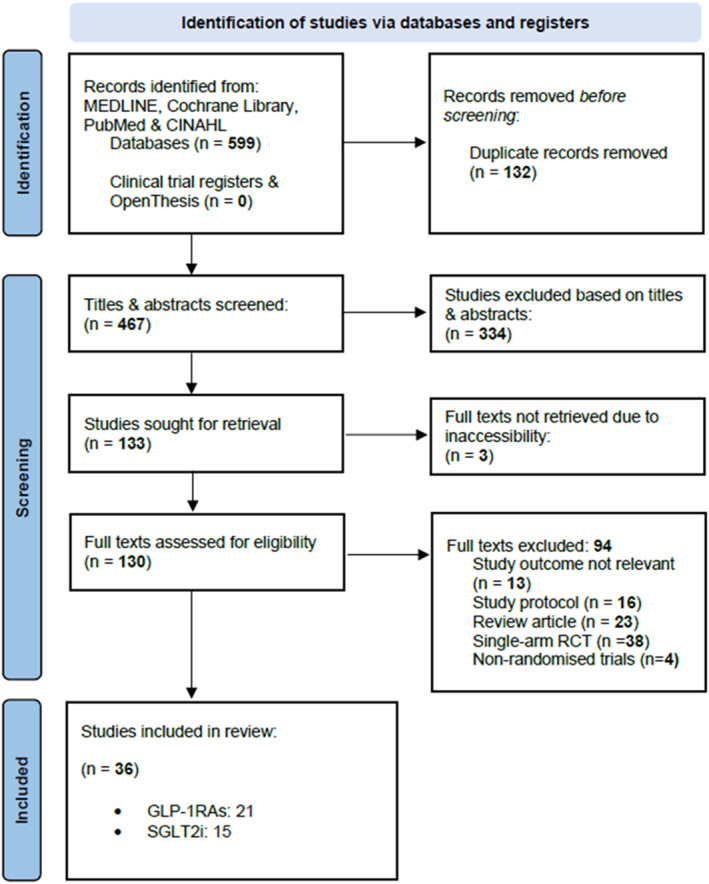
PRISMA flow‐chart of studies included.

Following our searches, a total of 599 articles were identified. After the removal of duplicates, 467 articles underwent screening by their title and abstract. 130 studies were screened by full text, from which 36 studies satisfied our inclusion criteria (see Figure 1). The studies involved a total of 5316 participants with a mean age ranging from 26.0 to 66.3 years. Men and women were recruited in 31 studies, with white ethnicity being most prevalent where reported. The length of included trials ranged from 8 to 102 weeks. Thirty‐four studies were randomised parallel group trials (RCTs) [7, 24, 25, 26, 27, 28, 29, 30, 31, 32, 33, 34, 35, 36, 37, 38, 39, 40, 41, 42, 43, 44, 45, 46, 47, 48, 49, 50, 51, 52, 53, 54, 55, 56]. Two were randomised cross‐over studies [57, 58]. Twenty studies were focused on those with T2DM [25, 27, 28, 29, 30, 31, 32, 33, 34, 40, 41, 45, 46, 47, 48, 53, 54, 55, 56, 57], eight were focused on wider populations with obesity [7, 24, 39, 42, 43, 44, 51, 52], five were focused on type 1 diabetes mellitus [35, 36, 37, 50, 58] and three were focused on polycystic ovarian syndrome [26, 38, 49]. In terms of pharmacotherapy, 21 studies investigated GLP‐1RAs [7, 24, 25, 26, 27, 28, 29, 30, 35, 37, 38, 39, 41, 42, 43, 46, 47, 50, 52, 57, 58] and 15 investigated SGLT2is [31, 32, 33, 34, 36, 40, 44, 45, 48, 49, 51, 53, 54, 55, 56]. No studies were included investigating dual GIP/GLP‐1RA agonists due to a lack of body composition data. For GLP‐1RAs, 15 studied the effect of liraglutide [24, 25, 26, 27, 28, 35, 37, 38, 39, 41, 42, 43, 46, 47, 58], four exenatide [29, 30, 50, 52] and two semaglutide [7, 57]. For SGLT2is, three studies investigated canagliflozin [31, 32, 44], eight dapagliflozin [33, 40, 45, 48, 51, 53, 55, 56], two ipragliflozin [34, 54], one sotagliflozin [36] and one empagliflozin [49]. For the control arms, 24 studies had a placebo arm [7, 24, 26, 27, 31, 33, 35, 36, 37, 38, 39, 41, 42, 43, 44, 45, 46, 47, 48, 50, 51, 52, 57, 58] and 12 studies had an active comparator arm or lifestyle intervention arm [25, 28, 29, 30, 32, 34, 40, 49, 53, 54, 55, 56], with the active comparator being another medication used for type 1 or type 2 diabetes such as; metformin, glimepiride, insulin glargine, glibenclamide, pioglitazone and tenegliptin. The characteristics of included GLP‐1RA studies are detailed in Table 1 and included SGLT2i studies in Table 2. The results for the risk of bias assessment for all included studies are given in the Supporting Information S1 (see Supporting Information S1: Figures S1 and S2). The risk of bias assessment for the 34 randomised parallel group trials had an overall moderate bias, and the two randomised cross‐over studies had an overall moderate bias (see Supporting Information S1: Table S1 and S2; Figures S1 and S2). All studies were deemed moderate quality by the GRADE system [59], and asymmetrical funnel plots suggested evidence of publication bias, with smaller studies reporting lower LBM loss (see Supporting Information S1: Figures S3 and S4).

**TABLE 1:** Studies reporting changes in lean body mass, fat‐free mass and skeletal muscle mass with GLP‐1RA therapy.

Study reference (first author and year if publication)	Trial length (weeks)	Study location	Total *n*	Population	Placebo/active comparator	Intervention (dose and frequency)	Measure of body composition	LBM change (placebo/active comparator group) mean change (kg) ± SD	LBM change (intervention group) (mean change [kg] ± SD)
Astrup et al. 2012 [24]	20	Europe (multi‐site)	72	Obesity	Placebo	Liraglutide 3.0 mg (once‐daily)	DEXA	−0.6 kg ± 3.7	−1.1 kg ± 4.3
Feng et al. 2019 [25]	24	China	58	T2DM	Metformin (1000 mg)	Liraglutide 1.8 mg (once‐daily)	DEXA	0.1 kg ± 9.7	−0.2 kg ± 8.8
Frossing et al. 2018 [26]	26	Denmark	72	PCOS	Placebo	Liraglutide 1.8 mg (once‐daily)	DEXA	0.1 kg ± 1.83	−2.4 kg ± 2.65
Harder et al. 2004 [27]	8	Denmark	33	T2DM	Placebo	Liraglutide 0.6 mg (once‐daily)	DEXA	−0.2 kg ± 6.9	0.6 kg ± 11.7
Jendle et al. 2009 [LEAD 2] [28]	26	Global (multi‐site)	57	T2DM	Placebo and metformin 1.5–2 mg)	Liraglutide 1.8 mg (once‐daily)	DEXA	−1.3 kg ± 1.65	−1.5 kg ± 5.88
Jendle et al. 2009 [LEAD 3] [28]	52	Global (multi‐site)	38	T2DM	Glimepiride (8 mg)	Liraglutide 1.8 mg (once‐daily)	DEXA	−0.6 kg ± 3.08	−1.5 kg ± 2.16
Bunck et al. 2010 [29]	52	Sweden	69	T2DM	Insulin glargine	Exenatide 20 μg (twice‐daily)	DEXA	0.5 kg ± 9.3	0.3 kg ± 12.2
Yin et al. 2018 [30]	16	China	37	T2DM	Insulin glargine	Exenatide 10 μg (twice‐daily)	DEXA	0.3 kg ± 5.7	−0.4 kg ± 9.4
Ghanim et al. 2020 [35]	26	USA	64	T1DM	Placebo	Liraglutide 1.8 mg (once‐daily)	DEXA	0.0 kg ± 11.2	−0.2 kg ± 13.1
Shmidt et al. 2022 [37]	26	Denmark	44	T1DM	Placebo	Liraglutide 1.8 mg (once‐daily)	DEXA	0.0 kg ± 1.57	−2.5 kg ± 1.57
Elkind‐Hirsch et al. 2022 [38]	32	USA	82	PCOS	Placebo	Liraglutide 3.0 mg (once‐daily)	DEXA	−0.3 kg ± 8.87	−0.9 kg ± 7.29
Wilding et al. 2021 [7]	68	Global (multi‐site)	140	Obesity	Placebo	Semaglutide 2.4 mg (once‐weekly)	DEXA	−1.83 kg ± 3.69	−5.26 kg ± 3.6
Neeland et al. 2021 [39]	46	USA	185	Obesity	Placebo	Liraglutide 3.0 mg (once‐daily)	MRI	−0.17 kg ± 5.93	−0.54 kg ± 7.5
van Eyk et al. 2020 [41]	26	Netherlands	49	T2DM	Placebo	Liraglutide 1.8 mg (once‐daily)	BIA	−0.2 kg ± 1.6	−2.1 kg ± 2.9
Kadouh et al. 2020 [42]	16	USA	40	Obesity	Placebo	Liraglutide 3.0 mg (once‐daily)	DEXA	−0.65 kg ± 1.05	−1.3 kg ± 0.93
Lundgren et al. 2021 [43]	52	Denmark	98	Obesity	Placebo	Liraglutide 3.0 mg (once‐daily)	DEXA	2.9 kg ± 3.8	0.0 kg ± 3.48
Gibbons et al. 2020 [57]	12	UK	15	T2DM	Placebo	Oral semaglutide 14 mg (per day)	ADP	0.5 kg ± 1.2	−0.1 kg ± 1.7
Mensberg et al. 2017 [46]	16	Denmark	36	T2DM	Placebo	Liraglutide 1.8 mg (once‐daily)	DEXA	0.7 kg ± 1.5	0.1 kg ± 2.2
van Eyk et al. 2019 [47]	26	Netherlands	47	T2DM	Placebo	Liraglutide 1.8 mg (once‐daily)	BIA	−0.2 kg ± 1.6	−2.3 kg ± 2.3
Dube et al. 2018 [58]	24	Canada	30	T1DM	Placebo	Liraglutide 1.8 mg (once‐daily)	BIA	−1.4 kg ± 3.5^a^	−2.3 kg ± 3.45^a^
Johansen et al. 2020 [50]	26	Denmark	105	T1DM	Placebo	Exenatide 10 μg (three times‐daily)	DEXA	−0.23 kg ± 1.71^a^	−1.38 kg ± 1.95^a^
Ishoy et al. 2017 [52]	14	Denmark	40	Obesity	Placebo	Exenatide 2 mg (weekly)	DEXA	−1.0 kg ± 7.8^b^	−0.7 kg ± 12.1^b^

Abbreviations: ADP, Air displacement plethysmography; BIA, Bio‐electrical impedance analysis; DEXA, Dual‐energy x‐ray absorptiometry; FFM, Fat‐free mass; LBM, Lean body mass; MRI, Magnetic resonance imaging; PCOS, Polycystic Ovary Syndrome; SD, Standard deviation; SMM, Skeletal muscle mass; T1DM, Type 1 Diabetes Mellitus; T2DM, Type 2 Diabetes Mellitus.

^a^ Data reported for fat‐free mass.

^b^ Data reported for skeletal muscle mass.

**TABLE 2:** Studies reporting changes in lean body mass, fat‐free mass and skeletal muscle mass with SGLT2i therapy.

Study reference (first author and year if publication)	Trial duration (weeks)	Study location	Total *n*	Population	Placebo/active comparator	Intervention (dose and frequency)	Measure of body composition	LBM change (placebo/active comparator group) mean change (kg) ± SD	LBM change (intervention group) (mean change ± SD)
Blonde et al. 2016 [study 2] [31]	26	Global (multi‐site)	148	T2DM	Placebo	Canagliflozin 300 mg oral (once‐daily)	DEXA	−0.3 kg ± 2.12	−1.2 kg ± 2.32
Cefalu et al. 2013 [32]	52	Global (multi‐site)	198	T2DM	Glimepiride 6 mg uptitrated to 8 mg as tolerated	Canagliflozin 300 mg oral (once‐daily)	DEXA	−1.2 kg ± 2.32	−1.1 kg ± 2.49
Bolinder et al. 2014 [33]	102	Europe (multi‐site)	137	T2DM	Placebo	Dapagliflozin 10 mg oral (once‐daily)	DEXA	−0.9 kg ± 2.11	−1.3 kg ± 2.03
Inoue et al. 2019 [34]	24	Japan	48	T2DM	Insulin	Ipragliflozin 50 mg oral (once‐daily)	DEXA	−0.3 kg ± 7.3	−0.6 kg ± 7.5
Rodbard et al. 2020 [36]	52	USA and Canada	52	T1DM	Placebo	Sotagliflozin 400 mg oral (once‐daily)	DEXA	0.43 kg ± 2.52	−0.39 kg ± 2.44
Wolf et al. 2021 [40]	12	Brazil	98	T2DM	Glibenclamide 5 mg + metformin	Dapagliflozin 10 mg oral (once‐daily)	DEXA	0.92 kg ± 1.17	−0.34 kg ± 1.36
Kashyap et al. 2020 [44]	26	USA	16	Obesity	Placebo	Canagliflozin 300 mg oral (once‐daily)	DEXA	−1.48 kg ± 4.05	0.92 kg ± 1.99
Bolinder et al. 2012 [45]	24	Europe (multi‐site)	180	T2DM	Placebo	Dapagliflozin 10 mg oral (once‐daily)	DEXA	−0.6 kg ± 14.2	−1.1 kg ± 33.5
Fadini et al. 2017 [48]	12	Italy	31	T2DM	Placebo	Dapagliflozin 10 mg oral (once‐daily)	BIA	0.1 kg ± 5.2^a^	−2.9 kg ± 5.03^a^
Javed et al. 2019 [49]	12	UK	39	PCOS	Metformin 1500 mg daily	Empagliflozin 25 mg oral (once‐daily)	BIA	−0.2 kg ± 7.9^a^	−1.1 kg ± 5.85^a^
Ryan et al. 2020 [51]	12	USA	57	Obesity	Placebo	Dapagliflozin 10 mg oral (once‐daily)	DEXA	−0.7 kg ± 11.3^a^	−1.9 kg ± 9.3^a^
Shimizu et al. 2019 [53]	24	Japan	57	T2DM	N/A	Dapagliflozin 5 mg oral (once‐daily)	BIA	−0.3 kg ± 5.21^b^	−0.9 kg ± 7.65^b^
Han et al. 2020 [54]	24	Korea	45	T2DM	Metformin + pioglitazone	Ipragliflozin 50 mg oral (once‐daily)	DEXA	0.3 kg ± 9.4^b^	−0.7 kg ± 11.8^b^
Yamakage et al. 2020 [55]	24	Japan	54	T2DM	Lifestyle intervention	Dapagliflozin 5 mg oral (once‐daily)	BIA	−0.2 kg ± 6.21^b^	0.1 kg ± 7.07^b^
Tobita et al. 2021 [56]	12	Japan	26	T2DM	Tenegliptin 20 mg/day	Dapagliflozin 5 mg oral (once‐daily)	BIA	0.0 kg ± 6.3^b^	−0.4 kg ± 9.2^b^

Abbreviations: BIA, Bio‐electrical impedance analysis; DEXA, Dual‐energy x‐ray absorptiometry; FFM, Fat‐free mass; LBM, Lean body mass; PCOS, Polycystic Ovary Syndrome; SD, Standard deviation; SMM, Skeletal muscle mass; T1DM, Type 1 Diabetes Mellitus; T2DM, Type 2 Diabetes Mellitus.

^a^ Data reported for fat‐free mass.

^b^ Data reported for skeletal muscle mass.

### 3.2 Body Composition Method

Twenty‐six studies adopted DEXA in measuring body composition [7, 24, 25, 26, 27, 28, 29, 30, 31, 32, 33, 34, 35, 36, 37, 38, 40, 42, 43, 44, 45, 46, 50, 51, 52, 54]. Eight studies adopted BIA [41, 47, 48, 49, 53, 55, 56, 58]. One study adopted MRI [39] and one study adopted ADP [57] (see Table 1).

### 3.3 Meta‐Analysis for GLP‐1RAs

**FIGURE 2:**
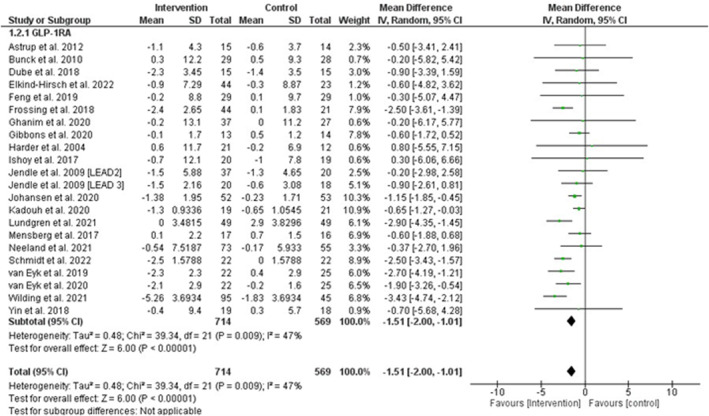
Weighted mean difference forest plot showing the effects of GLP‐1RAs on lean body mass. CI, confidence interval; IV, inverse variance.

Meta‐analysis of GLP‐1RAs (*n* = 21 studies) found an overall reduction in LBM in the direction of the intervention groups: (MD: −1.51 kg [95% CI: −2.00 to −1.01] *I* ^2^ = 47%) (see Figure 2). Results for the standardised mean difference were (SMD: −0.41 [95% CI: −0.60 to −0.23] *I* ^2^ = 60%; see Supporting Information S1: Figure S5).

### 3.4 Meta‐Analysis for SGLT2is

**FIGURE 3:**
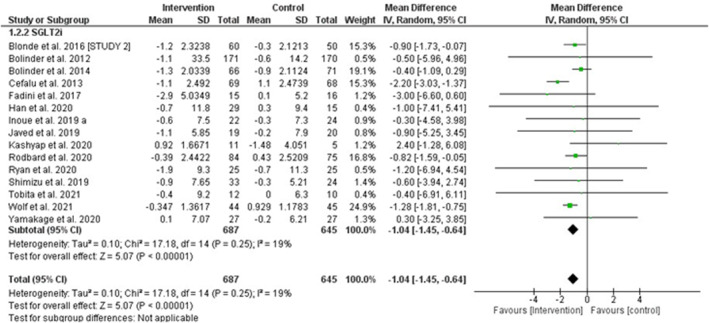
Weighted mean difference forest plot showing the effects of SGLT2is on lean body mass. CI, confidence interval; IV, inverse variance.

Meta‐analysis of SGLT2is (*n* = 15 studies) found an overall reduction of LBM in the direction of the intervention groups: (MD: −1.04 kg [95% CI: −1.45 to −0.64] *I* ^2^ = 19%; see Figure 3). Results for the standardised mean difference were (SMD: −0.27 [95% CI: −0.46 to −0.07] *I* ^2^ = 61%; see Supporting Information S1: Figure S6).

### 3.5 Meta‐Analysis Stratified by Body Composition Outcomes

Sub‐group analysis for GLP‐1RAs by body composition outcomes were LBM (*n* = 18 studies MD: −1.57 kg [95% CI: −2.14 to −0.99] *I* ^2^ = 53%); FFM (*n* = 2 studies MD: −1.13 kg [95% CI: −1.81 to −0.46] *I* ^2^ = 0%); SMM (*n* = 1 study MD: 0.30 kg [95% CI: −6.06 to 6.66] *I* ^2^ = N/A) (see Supporting Information S1: Figure S7). Results for SGLT2is were LBM (*n* = 8 studies MD: −1.02 kg [95% CI: −1.56 to −0.47] *I* ^2^ = 55%); FFM (*n* = 3 studies MD: −1.97 kg [95% CI: −4.46 to 0.53] *I* ^2^ = 0%); SMM (*n* = 4 studies MD: −0.29 kg [95% CI: −2.44 to 1.85] *I* ^2^ = 0%; see Supporting Information S1: Figure S8).

### 3.6 Meta‐Analysis Stratified by Body Composition Measurement Method

Sub‐group analysis for GLP‐1RAs by body composition method were DEXA (*n* = 16 studies MD: −1.54 kg [95% CI: −2.15 to −0.93] *I* ^2^ = 52%); BIA (*n* = 3 studies MD: −2.07 kg [95% CI: −3.01 to −1.14] *I* ^2^ = 0%); MRI (*n* = 1 study MD: −0.37 kg [95% CI: −2.70 to 1.96] *I* ^2^ = N/A); ADP (*n* = 1 study MD: −0.60 kg [95% CI: −1.72 to 0.52] *I* ^2^ = N/A; see Supporting Information S1: Figure S9). Results for SGLT2is were DEXA (*n* = 10 studies MD: −1.03 kg [95% CI: −1.53 to −0.53] *I* ^2^ = 42%); BIA (*n* = 5 studies MD: −0.99 kg [95% CI: −2.75 to 0.77] *I* ^2^ = 0%; see Supporting Information S1: Figure S10).

### 3.7 Meta‐Analysis Stratified by Disease Status

Sub‐group analysis for GLP‐1RAs by disease status were T2DM (*n* = 9 studies MD: −1.14 kg [95% CI: −1.72 to −0.57] *I* ^2^ = 0%); obesity (*n* = 6 studies MD: −1.62 kg [95% CI: −3.01 to −0.24] *I* ^2^ = 75%); PCOS (*n* = 2 studies MD: −2.38 kg [95% CI: −3.45 to −1.31] *I* ^2^ = 0%); T1DM (*n* = 4 studies MD: −1.62 kg [95% CI: −2.59 to −0.66] *I* ^2^ = 47%; see Supporting Information S1: Figure S11). Results for SGLT2is were T2DM (*n* = 11 studies MD: −1.14 kg [95% CI: −1.62 to −0.66] *I* ^2^ = 24%); obesity (*n* = 2 studies MD: 1.30 kg [95% CI: −1.95 to 4.55] *I* ^2^ = 7%); PCOS (*n* = 1 study MD: −0.90 kg [95% CI: −5.25 to 3.45] *I* ^2^ = N/A); T1DM (*n* = 1 study MD: −0.82 kg [95% CI: −1.59 to −0.05] *I* ^2^ = N/A; see Supporting Information S1: Figure S12).

### 3.8 Meta‐Analysis Stratified by the Percentage Proportion of LBM

**FIGURE 4:**
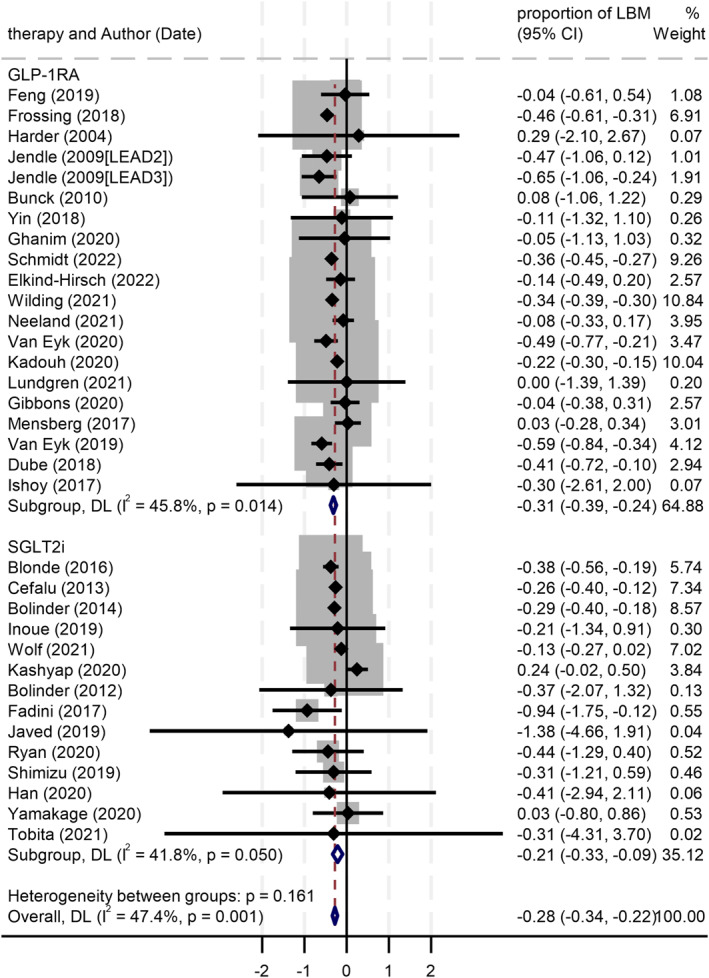
Forest plot showing the proportion of lean body mass contributing to overall weight change. Negative numbers represent the proportion of lean body mass in the context of overall weight loss.

Further analysis was conducted to investigate the proportion of overall weight loss that was attributable to LBM. Thirty‐three studies were included in this analysis; three studies were not included due to insufficient data. Sub‐group analysis stratified by therapy class found that 31% (95% CI: 24%–39%, *I* ^2^ = 45.8%) of overall weight loss from GLP‐1RAs resulted from LBM loss and 21% (95% CI: 9%–33%, *I* ^2^ = 41.8%) for SGLT2is (see Figure 4). Overall, across both classes of therapy 28% (95% CI: 22%–34%, *I* ^2^ = 47.4%) of weight loss was from LBM.

### 3.9 Meta‐Analysis Stratified by Comparison With Placebo or Active Control

Sub‐group analysis for GLP‐1RAs by comparator type were conducted for placebo‐controlled trials (*n* = 17 studies MD −1.61 kg, 95% CI −2.16 to −1.05; *p* < 0.00001; *I* ^2^ = 58%) and active comparator trials (*n* = 5 studies MD −0.65 kg, 95% CI −1.95 to 0.65; *p* = 0.33; *I* ^2^ = 0%; see Supporting Information S1: Figure S13). Sub‐group analysis for SGLT2is by comparator type were conducted for placebo‐controlled trials (*n* = 7 studies MD: −0.67 kg, 95% CI −1.10 to −0.24; *p* = 0.002; *I* ^2^ = 0%) and for active comparator trials (*n* = 8 studies MD: −1.47 kg, 95% CI −1.91 to −1.04; *p* < 0.00001; *I* ^2^ = 0%; see Supporting Information S1: Figure S14).

### 3.10 Meta‐Regression Analyses

Meta‐regression analyses were conducted to estimate the percentage sex distribution effect on LBM. The results showed that a higher percentage of women in the cohort led to an increased reduction in lean body mass (per kg); (co‐efficient: −0.013 [95% CI: −0.033 to 0.006]) per percentage difference, however the results were not significant (*p* = 0.183) (see Supporting Information S1: Figures S15 and S16 and Table S3). Additionally, meta‐regression analyses to estimate the effect of change in body weight (*p* = 0.921; Supporting Information S1: Figure S17 and Table S4), change in waist circumference (*p* = 0.648; Supporting Information S1: Figure S18 and Table S5) and mean age (*p* = 0.178; Supporting Information S1: Figure S19 and Table S6) on the percentage proportion of weight loss attributed of LBM were not significant.

## 4 Discussion

Our systematic review meta‐analysis found that there was a 1.51 kg loss in LBM with GLP‐1RA therapy and a 1.04 kg loss in LBM with SGLT2i therapy. Across both therapy classes, this equated to 28% of overall weight loss. Results were largely unaffected by the method of measurement for body composition, although there was some evidence trial design influenced the results with greater LBM loss observed in placebo controlled compared to active comparator trials for GLP‐1RA therapy, whereas the converse was observed for SGLT2i therapy.

While it is widely reported that weight loss in obesity, T2DM and associated metabolic disease are beneficial, these benefits may be limited by the loss of LBM [60]. GLP‐1RAs induce weight (and concomitantly LBM) loss by reducing caloric intake, driving negative protein‐energy balance [4, 5]. Conversely, glucosuria‐induced caloric loss following SGLT2i therapy contributes to LMB loss as muscle protein degradation is increased to release amino acids for gluconeogenesis [9]. Quantifying the loss of LBM is important, as it plays a key role in contributing to a broad range of physiological and metabolic processes, including energy balance [61]. LBM is also associated with glucose control by through affecting glucose storage capacity and lowering insulin sensitivity [62]. Although, where indicated, treatment with SGLT2is and GLP‐1RAs tends to result in a net improvement in glucose control, the potential loss of LBM may act to attenuate these effects, particularly in older adults or other high‐risk populations [8, 63]. Our findings extend another recent meta‐analysis that was published whilst the present study was ongoing, which reported a loss of 1.01 kg in skeletal muscle mass with SGLT2i therapy [64]. However, this previous analysis was restricted to SGLT2i therapy in populations with T2DM only, compared to the wider inclusion criteria reported in our meta‐analysis. Furthermore, our meta‐analysis includes GLP‐1RA therapies and reports LBM loss as percentage of overall weight loss. The latter analysis is important as it allows a comparison to the wider literature. For example, it suggested that both GLP‐1RA and SGLT2i therapies induce a proportional loss of LBM consistent with dietary interventions [65]. The loss of LBM with GLP‐1RAs and SGLT2is is therefore likely to be related to the induced weight loss and not further affected in a positive or negative direction by the specific mechanisms of the investigated pharmacotherapies. Emerging evidence for tirzepatide further supports this conclusion, with the proportion of lean mass loss being a third of that observed for fat mass (pooled sub‐study data not included in this meta‐analysis due to lack of body composition outcome data) [66].

Losses in LBM can result in reduced strength while accelerating the ageing process [67]. Previous research has shown that weight‐stable individuals lose an average of ≥ 1.5 kg of LBM per decade, therefore the LBM losses associated with GLP‐1RAs are consistent with a decade of biological ageing [68]. Although LBM loss through general weight loss may have different metabolic effects compared to LBM loss through ageing, it is likely that acting to attenuate the loss of LBM when undergoing weight loss will provide enhanced health benefits.

Studies have shown that combining multimodal exercise training with diet‐induced weight loss interventions are beneficial in preserving LBM, while enhancing cardiorespiratory fitness, improving physical function and metabolic health [69, 70, 71, 72]. Furthermore, a number of studies have investigated the combination of GLP‐1RAs and SGLT2is with exercise training on body composition and cardiorespiratory fitness [46, 73, 74]. Two studies [46, 73] investigating a combination with GLP‐1RAs implemented a minimum of two supervised 60‐min sessions per week, with one study including a specific session dedicated to resistance exercising training [46]. Both trials highlighted potential LBM preservation with weight loss along with improvements in cardiorespiratory fitness, with one study showing that combining exercise training with liraglutide led to an 8% reduced body weight primarily consisting of FM derived from visceral fat, while maintaining total LBM [73]. Conversely, a previous randomised controlled trial showed that SGLT2i therapy in combination with regular endurance exercise training did not attenuate or augment body mass, FM and LBM, whereas exercise training alone decreased FM while not changing LBM [74]. Given the limited evidence from previous research, there is an increased clinical need in understanding the synergistic effects between exercise interventions and pharmacotherapy on body composition and metabolic health.

### 4.1 Strengths and Limitations

Our meta‐analysis is the first to quantify the loss of LBM from weight loss induced by GLP‐1RAs. Further strengths include low heterogeneity amongst studies and the sub‐group sensitivity analysis undertaken based on therapy class, LBM, FFM and SMM and body composition measurement methods. Limitations include some trials having a small sample size with large cardiovascular and renal outcome trials for SGLT2is not included due to lack of body composition outcome data [75, 76, 77]. In addition, the latest dual agonist weight loss therapies were also not included due to a lack of complete body composition data within the main trial population [66]. Furthermore, although heterogeneity was generally low, differences in the study population, trial length, control arm consisting of placebo or active comparator and GLP‐1RA and SGLT2i dose variations may have increased heterogeneity in this meta‐analysis. Evidence of publication bias also makes it challenging to draw definitive conclusions. Additionally, as the longest follow‐up in the analysis was 102 weeks, the longer‐term effects of GLP‐1RAs and SGLT2is on LBM are not fully known. Most studies did not report data for ethnic groups, therefore there is a need to investigate how prescribed GLP‐1RA and SGLT2i therapies affect LBM loss in different ethnic populations. This is of particular importance in South Asian ethnic minority groups, who are known to have lower levels of LBM, cardiorespiratory fitness and a greater risk of frailty compared to a white population, therefore LBM preservation is a resulting priority [78].

## 5 Conclusion

This systematic review and meta‐analysis demonstrates that GLP‐1RAs lead to a loss of 1.5 kg in LBM whereas SGLT2is lead to a loss of 1 kg in LBM. Future research with newer generations of GLP‐1RA therapies that promote greater weight loss is warranted, in addition to strategies that preserve and increase LBM in patient groups prescribed weight loss therapies. A particular emphasis must be placed on investigating the benefits of combining structured exercise training with pharmacotherapy in clinical practice.

## Author Contributions

T.Y., J.A.S. and R.J. conceived the research question. T.Y., J.A.S. and R.J. developed the study methodology. R.J., T.Y., E.J. and J.A.S. contributed to the drafting of the manuscript. R.J., A.A. and E.J. conducted the study screening and risk of bias assessment. R.J. and E.J. conducted the data extraction. R.J., E.J. and J.A.S. conducted the data analysis. All authors assisted in the revising of the manuscript.

## Conflicts of Interest

R.J., E.J., T.P. and A.A. report no conflict of interest. J.A.S. has received a grant in support of an investigator‐initiated trial from AstraZeneca. D.R.W. has received honoraria as a speaker for AstraZeneca, Sanofi‐Aventis and Lilly, and has received research funding support from Novo Nordisk. M.J.D. has acted as consultant, advisory board member and speaker for Novo Nordisk, Sanofi‐Aventis, Lilly, Merck Sharp & Dohme, Boehringer Ingelheim, AstraZeneca and Janssen, an advisory board member for Lexicon, Servier and Gilead Sciences Ltd and as a speaker for Napp Pharmaceuticals, Mitsubishi Tanabe Pharma Corporation and Takeda Pharmaceuticals International Inc. She has received grants in support of investigator and investigator‐initiated trials from Novo Nordisk, Sanofi‐Aventis, Lilly, Boehringer Ingelheim, AstraZeneca and Janssen. T.Y. is supported by the NIHR Leicester BRC and has received funding from Astra Zeneca for an investigator‐initiated project.

## Supporting information


Supporting Information S1

## Data Availability

No new data were generated or analyzed in this study. All data used in this meta‐analysis are derived from previously published studies and are cited within the article.
