# Supplementary material for: The Effects of Glucagon‐Like Peptide‐1 Receptor Agonists and Sodium‐Glucose Co‐Transporter‐2 Inhibitors on Lean Body Mass in Humans: A Systematic Review and Meta‐Analysis of Randomised Controlled Trials
Source: Diabetes Metab Res Rev. 2026 Jun 19;42(5):e70194. doi: 10.1002/dmrr.70194 (PMC13282017; doi:10.1002/dmrr.70194)
Supplement: Supplementary file 1 — Supporting Information S1 [file DMRR-42-e70194-s001.docx]

**Supplementary materials**

**The effects of glucagon-like** **peptide-1 receptor agonists and sodium-glucose co-transporter 2 inhibitors on lean body mass in humans: A systematic review and meta-analysis of randomised controlled trials**

***Supplementary Methods 1***

***MEDLINE Search strategy***

1 exp Weight Loss/ 48213

2 exp Body Weight/ 516334

3 weight reduc*.mp. 14730

4 1 or 2 or 3 520949

5 exp Body Composition/ 62280

6 dual-energy x-ray absorptiometry.mp. 23207

7 DEXA.mp. 4733

8 Skinfold Thickness.mp. 8473

9 skinfold caliper test.mp. 0

10 bodpod.mp. 94

11 hydrostatic weighing.mp. 411

12 impedance.mp. 68764

13 exp lean muscle mass/ 0

14 exp body fat percentage/ 0

15 exp fat mass/ 0

16 exp fat free mass/ 0

17 exp total body water/ 0

18 5 or 6 or 7 or 8 or 9 or 10 or 11 or 12 or 13 or 14 or 15 or 16 or 17 149267

19 glucagon-like peptide-1 receptor agonists.mp. 1874

20 GLP-1RAs.mp. 508

21 exp Sodium-Glucose Transporter 2 Inhibitors/ 5174

22 dual GLP-1 GIP receptor agonists.mp. 13

23 exp Liraglutide/ 2326

24 exp Exenatide/ 2816

25 semaglutide.mp. 924

26 ozempic.mp. 16

27 dulaglutide.mp. 626

28 trulicity.mp. 22

29 efpeglenatide.mp. 27

30 langlenatide.mp. 1

31 albiglutide.mp. 230

32 eperzan.mp. 4

33 tanzeum.mp. 9

34 exp Canagliflozin/ 903

35 dapagliflozin.mp. 2289

36 farxiga.mp. 34

37 forxiga.mp. 34

38 empagliflozin.mp. 2367

39 jardiance.mp. 47

40 ipragliflozin.mp. 283

41 suglat.mp. 5

42 luseogliflozin.mp. 137

43 lusefi.mp. 2

44 tofogliflozin.mp. 148

45 deberza.mp. 1

46 ertugliflozin.mp. 225

47 steglatro.mp. 16

48 sotagliflozin.mp. 139

49 zynquista.mp. 2

50 tirzepatide.mp. 125

51 mounjaro.mp. 5

52 19 or 20 or 21 or 22 or 23 or 24 or 25 or 26 or 27 or 28 or 29 or 30 or 31 or 32 or 33 or 34 or 35 or 36 or 37 or 38 or 39 or 40 or 41 or 42 or 43 or 44 or 45 or 46 or 47 or 48 or 49 or 50 or 51 14544

53 4 and 18 and 52

| **Study ID** | **D1** | **D2**  **Supplementary Table 1:** Risk of bias assessment for the randomised parallel group trials. | **D3** | **D4** | **D5** | **Overall** |
| --- | --- | --- | --- | --- | --- | --- |
| Astrup et al. 2012 |  |  |  |  |  |  |
| Feng et al. 2019 |  |  |  |  |  |  |
| Frossing et al. 2018 |  |  |  |  |  |  |
| Harder et al. 2004 |  |  |  |  |  |  |
| Jendle et al. 2009 |  |  |  |  |  |  |
| Bunck et al. 2010 |  |  |  |  |  |  |
| Yin et al. 2018 |  |  |  |  |  |  |
| Blonde et al. 2016 |  |  |  |  |  |  |
| Cefalu et al. 2013 |  |  |  |  |  |  |
| Bolinder et al. 2014 |  |  |  |  |  |  |
| Inoue et al. 2019 |  |  |  |  |  |  |
| Ghanim et al. 2020 |  |  |  |  |  |  |
| Rodbard et al. 2020 |  |  |  |  |  |  |
| Schmidt et al. 2022 |  |  |  |  |  |  |
| Elkind-Hirsch et al. 2022 |  |  |  |  |  |  |
| Wilding et al. 2021 |  |  |  |  |  |  |
| Neeland et al. 2021 |  |  |  |  |  |  |
| Wolf et al. 2021 |  |  |  |  |  |  |
| van Eyk et al. 2020 |  |  |  |  |  |  |
| Kadouh et al. 2020 |  |  |  |  |  |  |
| Lundgren et al. 2021 |  |  |  |  |  |  |
| Kashyap et al. 2020 |  |  |  |  |  |  |
| Bolinder et al. 2012 |  |  |  |  |  |  |
| Mensberg et al. 2017 |  |  |  |  |  |  |
| van Eyk et al. 2019 |  |  |  |  |  |  |
| Fadini et al. 2017 |  |  |  |  |  |  |
| Javed et al. 2019 |  |  |  |  |  |  |
| Johansen et al. 2020 |  |  |  |  |  |  |
| Ryan et al. 2020 |  |  |  |  |  |  |
| Ishoy et al. 2017 |  |  |  |  |  |  |
| Shimizu et al. 2019 |  |  |  |  |  |  |
| Han et al. 2020 |  |  |  |  |  |  |
| Yamakage et al. 2020 |  |  |  |  |  |  |
| Tobita et al. 2021 |  |  |  |  |  |  |

|  | \|  \| \| --- \| | Low risk |  |  |
| --- | --- | --- | --- | --- | --- |
|  |  | Some concerns |  |  |
|  |  | High risk |  |  |
|  |  |  |  |  |
|  | D1 | Randomisation process |  |  |
|  | D2 | Deviations from the intended interventions |  |  |
|  | D3 | Missing outcome data |  |  |
|  | D4 | Measurement of the outcome |  |  |
|  | D5 | Selection of the reported result |  |  |

| **Study ID** | **D1** | **DS** | **D2** | **D3** | **D4** | **D5** | **Overall** |
| --- | --- | --- | --- | --- | --- | --- | --- |
| Gibbons et al. 2020 |  |  |  |  |  |  |  |
| Dube et al. 2018 |  |  |  |  |  |  |  |

**Supplementary Table 2:** Risk of bias assessment for the randomised cross-over trials.

|  | \|  \| \| --- \| | Low risk |  |  |
| --- | --- | --- | --- | --- | --- |
|  |  | Some concerns |  |  |
|  |  | High risk |  |  |
|  |  |  |  |  |
|  | D1 | Randomisation process |  |  |
|  | D2 | Deviations from the intended interventions |  |  |
|  | D3 | Missing outcome data |  |  |
|  | D4 | Measurement of the outcome |  |  |
|  | D5 | Selection of the reported result |  |  |

**Supplementary Figure 1:** Risk of bias graph for randomised parallel group trials.

**Supplementary Figure 2:** Risk of bias graph for the randomised cross-over trials.


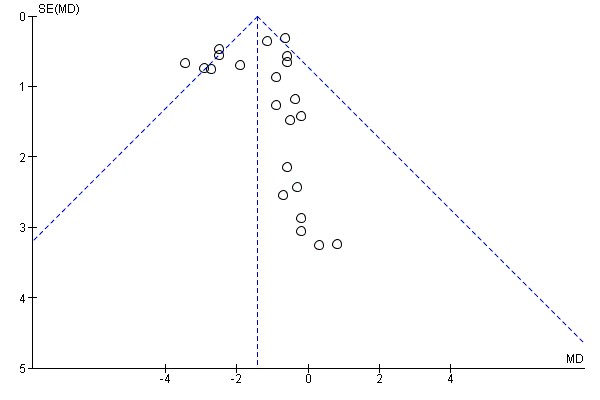


**Supplementary Figure 3:** Funnel plot highlighting the publication bias for GLP-1RA included studies.


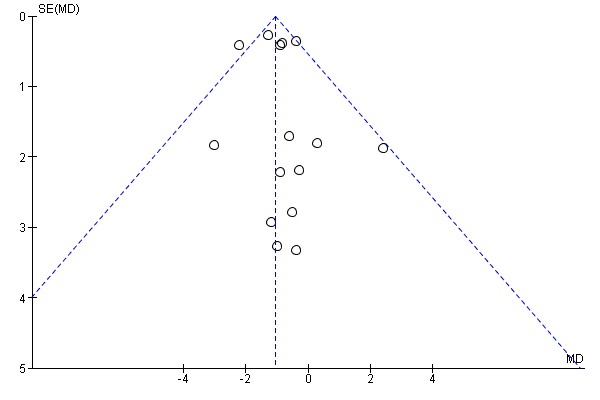


**Supplementary Figure 4:** Funnel plot highlighting the publication bias for SGLT2i included studies.


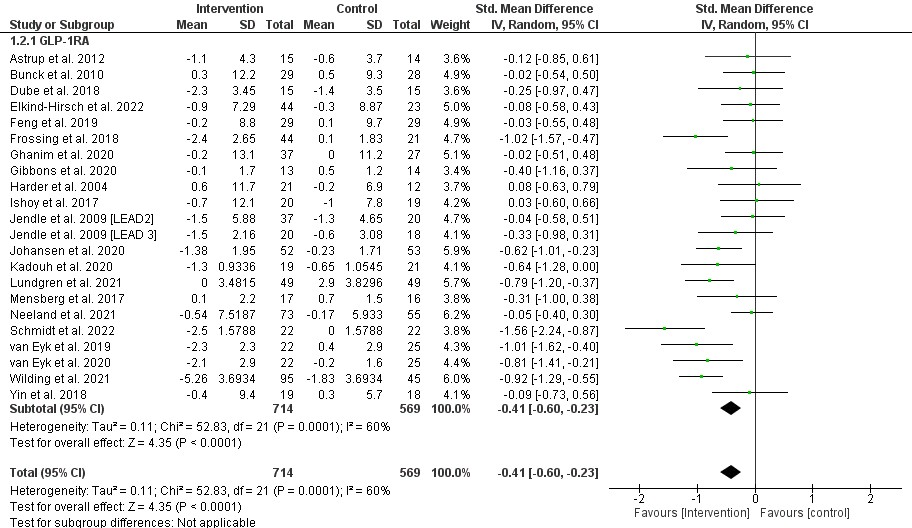


**Supplementary Figure 5:** Standardised mean difference (SMD) forest plot showing the effects of GLP-1RAs on lean body mass. Abbreviations: confidence interval (CI), inverse variance (IV).


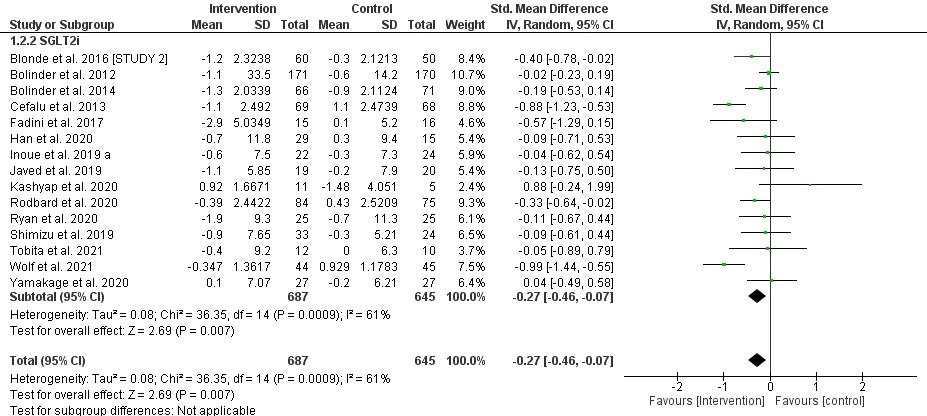


**Supplementary Figure 6:** Standardised mean difference (SMD) forest plot showing the effects of SGLT2is on lean body mass. Abbreviations: confidence interval (CI), inverse variance (IV).


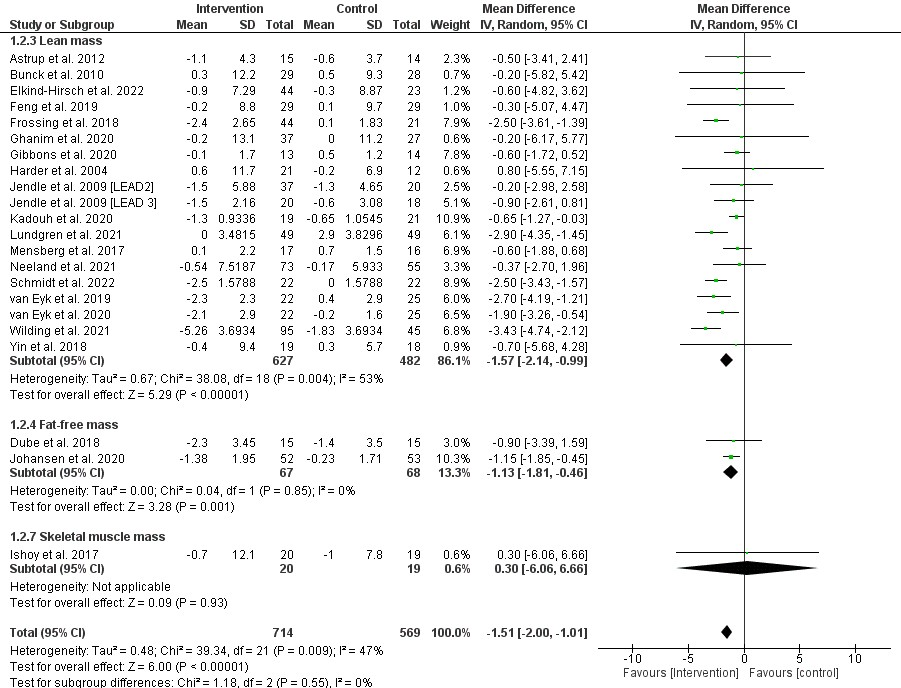


**Supplementary Figure 7:** Weighted mean difference forest plot with sub-group analysis for GLP-1Ras, stratified by body composition outcomes of interest, lean body mass, fat-free mass and skeletal muscle mass. Abbreviations: confidence interval (CI), inverse variance (IV).


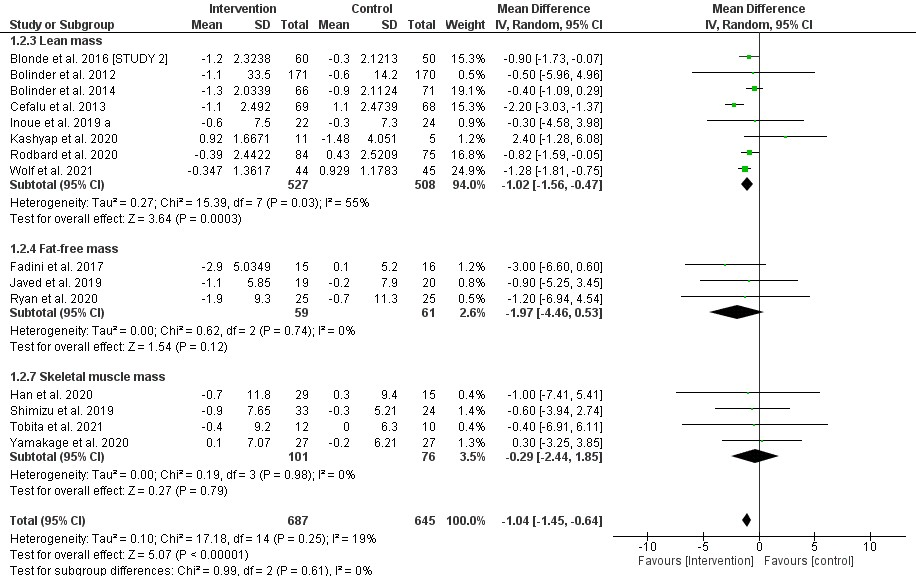


**Supplementary Figure 8:** Weighted mean difference forest plot with sub-group analysis for SGLT2is, stratified by body composition outcomes of interest, lean body mass, fat-free mass and skeletal muscle mass. Abbreviations: confidence interval (CI), inverse variance (IV).


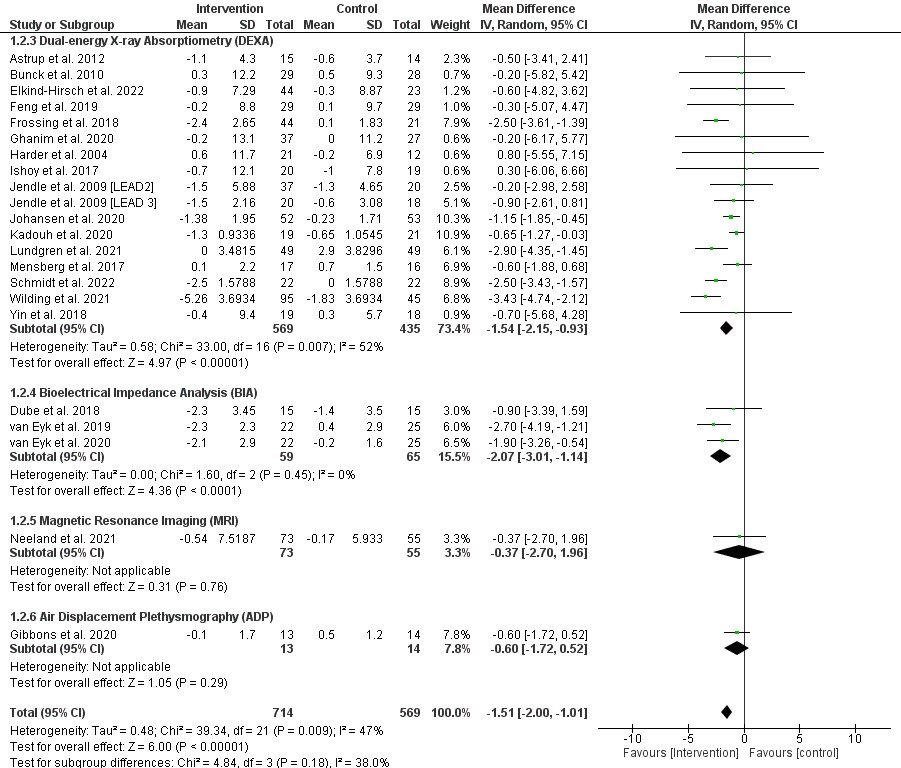


**Supplementary Figure 9:** Weighted mean difference forest plot with sub-group analysis for GLP-1Ras, stratified by body composition measurement method, dual-energy x-ray absorptiometry, bioelectrical impedance analysis, magnetic resonance imaging and air displacement plethysmography. Abbreviations: confidence interval (CI), inverse variance (IV).


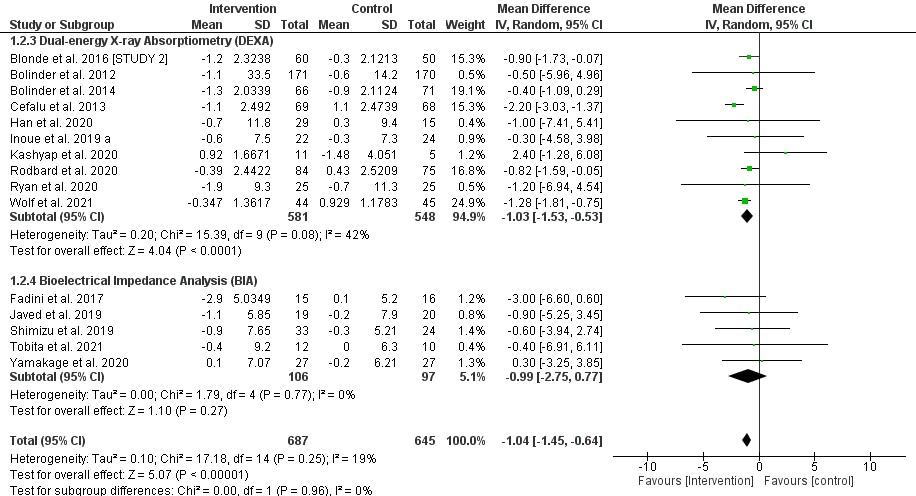


**Supplementary Figure 10:** Weighted mean difference forest plot with sub-group analysis for SGLT2is, stratified by body composition measurement method, dual-energy x-ray absorptiometry, bioelectrical impedance analysis, magnetic resonance imaging and air displacement plethysmography. Abbreviations: confidence interval (CI), inverse variance (IV).


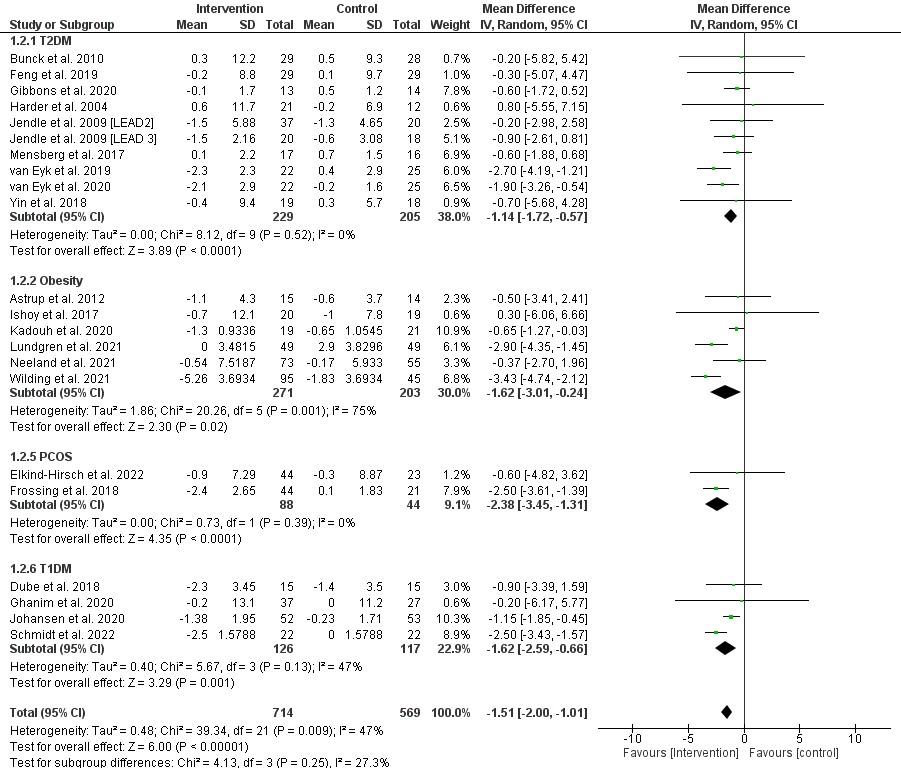


**Supplementary Figure 11** Weighted mean difference forest plot with sub-group analysis for GLP-1Ras, stratified by disease status, type 2 diabetes mellitus, obesity, polycystic ovary syndrome and type 1 diabetes mellitus. Abbreviations: confidence interval (CI), inverse variance (IV).


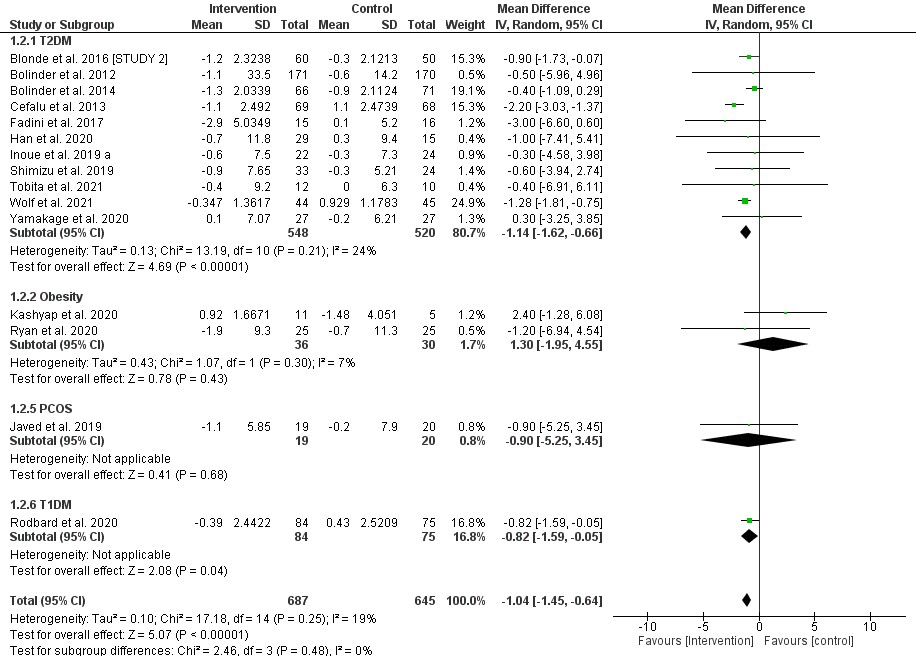


**Supplementary Figure 12:** Weighted mean difference forest plot with sub-group analysis for SGLT2is, stratified by based on disease status, type 2 diabetes mellitus, obesity, polycystic ovary syndrome and type 1 diabetes mellitus. Abbreviations: confidence interval (CI), inverse variance (IV).

**
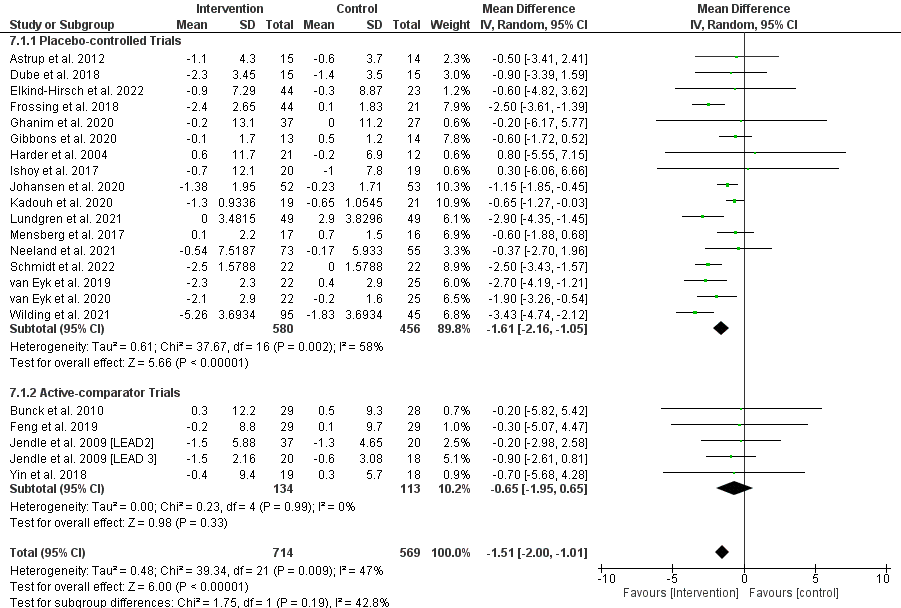
**

**Supplementary Figure 13:** Weighted mean difference forest plot with sub-group analysis for GLP-1Ras, stratified by comparison with placebo or active comparator. Abbreviations: confidence interval (CI), inverse variance (IV).

**
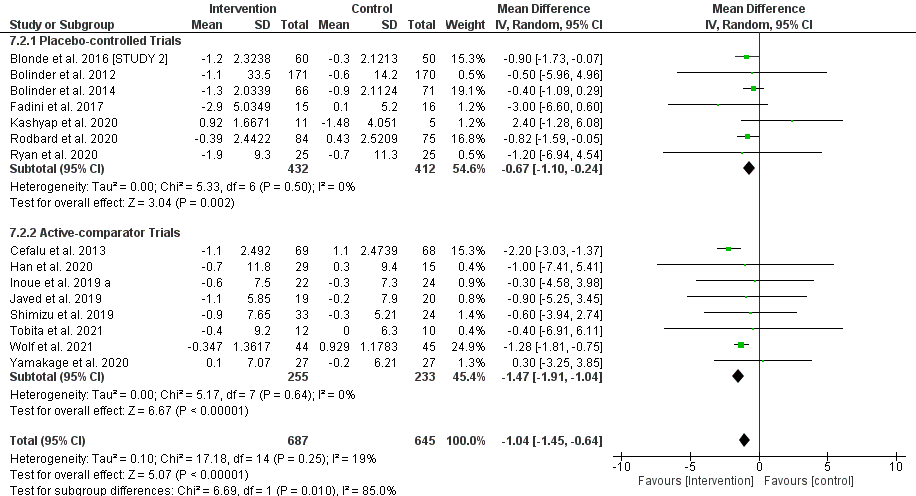
**

**Supplementary Figure 14:** Weighted mean difference forest plot with sub-group analysis for SGLT2is, stratified by comparison with placebo or active comparator. Abbreviations: confidence interval (CI), inverse variance (IV).


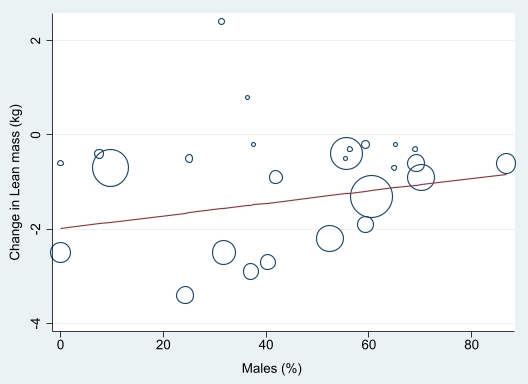


Men

**Supplementary Figure 15:** Meta-regression graph showing the percentage sex distribution of men and the effect on lean body mass.


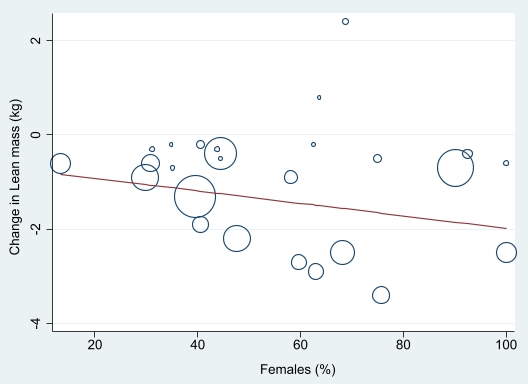


Women

**Supplementary Figure 16:** Meta-regression graph showing the percentage sex distribution of women and the effect on lean body mass.


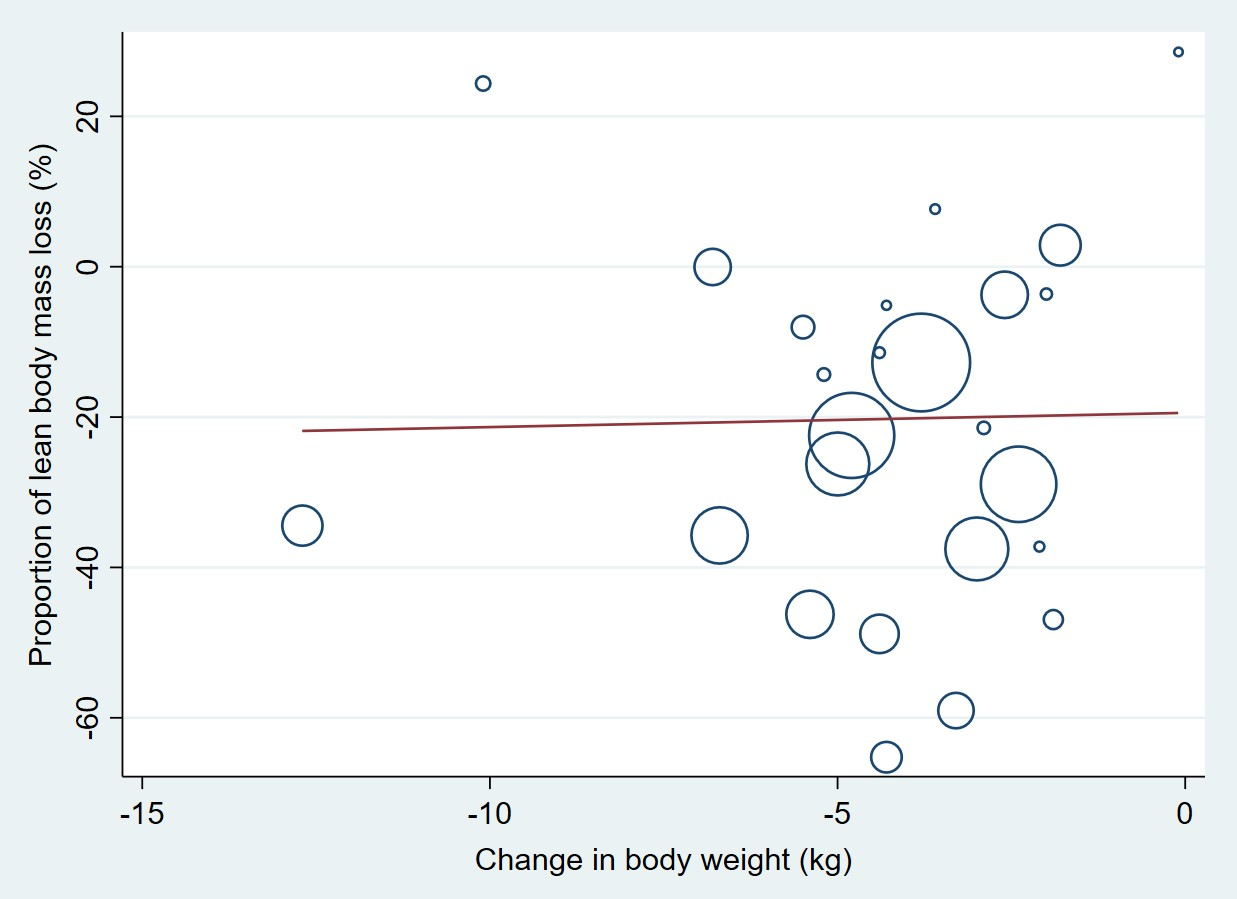


**Supplementary Figure 17:** Meta-regression graph showing the effect of change in body weight on the percentage proportion of lean body mass loss.

**Supplementary Figure 18:** Meta-regression graph showing the effect of change in waist circumference on the percentage proportion of lean body mass loss.

**Supplementary Figure 19:** Meta-regression graph showing the effect of mean age on the percentage proportion of lean body mass loss.

**Supplementary Table 3:** Meta regression analysis results showing the percentage sex distribution effect on lean body mass.

| Study variables | | Coefficient (95% CI) | *P* value | I^2^ (%) |
| --- | --- | --- | --- | --- |
| Men  Women | 0.013 (-0.006 to 0.033)  -0.013 (-0.033 to 0.006) | | 0.183  0.183 | 54.53  54.53 |

**Abbreviations:** CI: Confidence interval.

**Supplementary Table 4:** Meta regression analysis results showing the effect of the change in body weight on the percentage proportion of lean body mass loss.

| Study variables | Coefficient (95% CI) | | *P* value | I^2^ (%) |
| --- | --- | --- | --- | --- |
| Change in body weight on the proportion of LBM loss (%) | | 0.189 (-3.707 to 4.086) | 0.921 | 99.86 |

**Abbreviations:** LBM: lean body mass, CI: Confidence interval.

**Supplementary Table 5:** Meta regression analysis results showing the effect of the change in waist circumference on the percentage proportion of lean body mass loss.

| Study variables | Coefficient (95% CI) | | *P* value | I^2^ (%) |
| --- | --- | --- | --- | --- |
| Change in waist circumference on the proportion of LBM loss (%) | | -1.419 (-7.849 to 5.010) | 0.648 | 99.99 |

**Abbreviations:** LBM: lean body mass, CI: Confidence interval.

**Supplementary Table 6:** Meta regression analysis results showing the effect of mean age on the percentage proportion of lean body mass loss.

| Study variables | Coefficient (95% CI) | | *P* value | I^2^ (%) |
| --- | --- | --- | --- | --- |
| Mean age (years) on the proportion of LBM loss (%) | | 0.795 (-0.383 to 1.973) | 0.178 | 99.99 |

**Abbreviations:** LBM: lean body mass, CI: Confidence interval.
